# Global Gene Expression of *Kosteletzkya virginica* Seedlings Responding to Salt Stress

**DOI:** 10.1371/journal.pone.0124421

**Published:** 2015-04-22

**Authors:** Xiaoli Tang, Hongyan Wang, Chuyang Shao, Hongbo Shao

**Affiliations:** 1 Key Laboratory of Coastal Biology & Bioresources Utilization, Yantai Institute of Coastal Zone Research (YIC), Chinese Academy of Sciences (CAS), Yantai, 264003, China; 2 Institute of Biotechnology, Jiangsu Academy of Agricultural Sciences, Nanjing, 210014, China; 3 Yantai Academy of China Agriculture University, Yantai, 264670, China; 4 Shandong Agricultural University, Taian, Shandong, 271018, China; 5 University of Chinese Academy of Sciences, Beijing, 100049, China; National Taiwan University, TAIWAN

## Abstract

Soil salinization is becoming a serious threat to crop yield all over the world. Nowadays, acquainting the specific molecular mechanisms underlying various abiotic stresses especially to salt stress should be of great importance. While the development of the high-throughout sequencing technology promoted the progress powerfully. The intricate perception, transduction and regulation mechanisms underlying salt stress are being illustrated more and more clearly. As a perennial halophytic plant, *Kosteletzkya virginica* is able to help us to understand the mechanisms more directly and effectively. We carried out the whole transcriptome analysis on young seedlings with or without salt treatment through high-throughout sequencing technology. The results revealed that the numbers of different expressed transcripts between control and different treatments are 4145 and 9134, respectively. The ORF prediction suggested that there were 94308 ORF out of the 103489 (91.10%) total transcripts. We also carried out further differential expression analysis through gene ontology (GO) classification, cluster of orthologous groups (COG) and Kyoto Encyclopedia of Genes and Genomes (KEGG) analysis. In a word, our transcriptome study on *Kosteletzkya virginica* would provide direct and effective references for researches on molecular mechanisms of salt-tolerance, extending our view of salt tolerance in plant further. Above all, the related report in this paper is the first about *Kosteletzkya virginica*.

## Introduction

Soil salinity is becoming a critical challenge to agriculture worldwide rather than an agonizing problem merely in arid and semi-arid regions. FAO has reported that as much as 6% of the total world land is subjected to salinity [1]. Thus, the salt stress caused considerable crop yield losses and restricted the advances in agriculture [2]. More seriously, the condition is compounded by the growing agricultural need to provide enough nutrition for the world population, which is increasing steadily [3]. Confronted with the serious challenge, scientists are sparing no efforts to work out and the researches on plant science have assisted us a lot to enhance food security and agricultural sustainability [4]. Several decades ago, strategies for use of the salt-affected land including reclamation of the salt-affected soils, cultivation halophytes and breeding salt-tolerant crops have been proposed [2]. On the other hand, to counteract the disadvantageous circumstance, plants during the lengthy evolution have acquired many biochemical and molecular mechanisms. For example, the biochemical strategies are the synthesis of compatible solutes, control of ion uptake, induction of oxidative enzymes, synthesis of phytohormones and so on [5] As far as we know the molecular mechanisms are far more complex. Only the related transcription factors contain basic leucine zipper (bZIP) [6], WRKY [7], MYB [8] basic helix-loop-helix (bHLH) [9] and NAC family [10]. Not to mention the genes, proteins and processes regulated by these transcription factors. It was reported recently that most of the transcriptional changes induced by high salinity occur at approximately 3h after salt treatment [2]. More over the regulation of the phytohormones such as ABA, JA, BR and GA constitutes another vital and intricate component. Therefore, to be able to obtain the salt-tolerant crops, we must realize the concrete mechanisms and tactics and take full advantages of them.

*Kosteletzkya virginica*, also generally known as seashore mallow (SM), is a perennial halophytic species originated from the mid-Atlantic coasts and Southeastern of the US [11]. Duo to its doughty salt tolerance and potential ecological value, it was introduced as an important salt-resistant oil crop species to Jiangsu province, China in 1992 [12]. Naturally, the plant height of *Kosteletzkya virginica* is able to achieve 0.6–1.8 m and produces pink blooms during flowering phase. It is a suitable feedstock for the production of biodiesel and ethanol with 22% oil content in seed [13]. However, the foremost merit of *Kosteletzkya virginica* is its strong salt resistance. It was reported that it was able to complete growth and development normally in soil with 0.3 to 2.5% sodium salt (mainly NaCl) [14]. It is because of this characteristic that its performances under different concentrations of NaCl treatments in different developmental stages and tissues at physiological level are studied widely [2,15]. All the researches demonstrate that it is indeed a halophytic plant and is able to act as a model for the exploration of plant resistance. More importantly it is also a genetic resource to serve for our salt-tolerant crops breeding.

Recently, to understand and decode the transcriptome complexity and the possible functionalities of novel genes have become the primary mission of post-genome era [16]. In order to clarify the complicated transcripts, scientists have developed a series of classical methods like Northern blot, reverse transcriptase PCR, rapid amplification of cDNA ends (RACE), microarrays and so on [17]. Matsui et al. analyzed the *Arabidopsis* transcriptome under drought, cold, high-salinity and ABA treatments through tilling array [18]. Also in *Arabidopsis*, the expressions of as many as 5590 genes were discovered to be influenced by salt treatment [19]. With the rapid development of the sequencing technique, next generation sequencing (or RNA-seq) has revolutionized our understanding of transcriptomics. It possesses several wonderful advantages over the classical methods, for example, it does not need the previous genome annotation or nucleic acid probes [20]. It is able to reconstruct the transcriptome sequence information through *de novo* assembling the millions of sequenced reads instead of depending on the reference-based assembly strategies [21]. At present, the next generation sequencing mainly includes the Roche/454 Genome sequencer FLX, the ABI SOLiD System and the Illumina Genome analyzer [22,23]. This technology has been used for numerous prokaryotes and eukaryotes, model and non-model organisms to reveal their entire transcriptome dynamically and quantitatively [24]. More importantly, *de novo* transcriptome sequencing of some plants such as *Betula* [2], *Lupinus albus* (lupin) [2], *Cicer arietinum* (chickpea) [25], *Ipomoea batatas* (sweet potato) [26], *Chlorophytum borivilianum* [27], *Sorghum bicolor*[28] and *Medicago sativa* (alfalfa) [2] has already completed. With the popularity of next generation sequencing (NGS), the relationship between genotype and phenotype is being illustrated with greater resolution therefore it is a promising tool to help us to foster crops with favorable traits [29].

Studies on plant responses to abiotic stress emerge in endlessly especially for high salinity stress. The intricate perception, transduction and regulation mechanisms are being illustrated more and more clear. However most of the researches only focus on model plants and the reason is that we know more about them such as *Arabidopsis*, tobacco and rice. Their known genome sequences make our research simple and convenient, yet as a matter of fact they are not halophyte or halobiotic species and unsuitable for the exploration of special salt resistance mechanisms and genes. To comprehensively understand more about the responses and discover novel proteins or genes related to salt resistance, we adopted the high-throughput screening technique to sequence the transcriptome of the non-model but halophytic plant *Kosteletzkya virginica*. We investigated the gene expression profiles of *Kosteletzkya virginica* seedlings under salt stress. Here in this paper we will describe the results of a comparative transcriptome analysis of NaCl-treatment seedlings through RNA-seq. By comparing the different genes appeared in control group and treatment groups, we can learn more about the responses employed by *Kosteletzkya virginica* and find novel associated genes or strategies in salt resistant species. The objective of our research is to construct the first halobiotic plant transcriptome and to go more deep into to recognize the salt tolerance and resistance induced by high salinity in *Kosteletzkya virginica* seedlings for the first time. Meanwhile, our study of transcriptome will provide a large amount of gene sequence information and lay the foundation for further investigation.

## Materials and Methods

### STATEMENT: The field belongs to our Institute (YIC-CAS), and it is naturally permitted

#### Plant material and NaCl treatment

The seeds were obtained from the Eco-monitoring Station of YIC-CAS, the Yellow River Delta, Shandong Province, China and sterilized in 70% alcohol solution for 30 s, 0.1% solution of mercuric chloride for 10 min. Then the disinfectant seeds were washed by sterile distilled water thoroughly. The seeds were fostered in moderate liquid MS at 25°C in darkness for germination and transferred to plastic pots with perlite and vermiculite for further growth and development. The seedlings were cultivated under 16h light/8h dark with 25/18°C in artificial climatic chambers (Huier, China) and the humidity was kept at 65%.

The two-week old homogeneous seedlings were selected for experimental research and the materials were divided into triplet. One for control and the others were for NaCl treatment. Salt treatments were carried out by irrigating 1/2 Hoagland with concentration of 300 mM NaCl and the duration were 2 and 12 hours respectively. Once the treatments were finished, the seedlings were harvest, washed, dried and quick-frozen in liquid nitrogen. The refrigerant samples were preserved in -80°C for the following experiments.

#### RNA preparation, Library preparation and Illumina sequencing

For RNA-seq the total RNA were extracted from *Kosteletzkya virginica* young seedlings with Trizol Reagent (Invitrogen Carlsbad, CA, USA). The quality and quantity of the RNA were detected by Agilent 2100 BioAnalyzer (Agilent Technologies, CA, USA). For mRNA library construction and deep sequencing, RNA samples were prepared by using the TruSeq RNA Sample Preparation Kit according to the manufacture’s protocol. Briefly, the poly-A containing mRNA molecules were purified from the 4 μg of total RNA by using poly-T oligo-attached magnetic beads using two rounds of purification. During the second elution of the poly-A RNA, the RNA was fragmented using the divalent cations under 95°C. The cleaved RNA fragments were reversely transcribed into first strand cDNA using random hexamers, following by second strand cDNA synthesis using DNA Polymerase I and RNase H. The cDNA fragments were performed end repair process to convert the overhangs into blunt ends by an End Repair (ERP) mix. The 3’ to 5’ exonuclease activity of this mix removes the 3’ overhangs and the polymerase activity fills in the 5’ overhangs. A single ‘A’ nucleotide was then added to the 3’ends of the blunt fragments to prevent them from linking to one another during the adapter ligation reaction. A corresponding single ‘T’ nucleotide on the 3’ end of the adapter provides a complementary overhang for connecting the adapter to the fragment. This strategy ensured a low rate of chimera (concatenated template) formation. The multiple indexing adapters were linked to the ends of the ds cDNA, preparing them for hybridization onto a flow cell. PCR was used to selectively enrich those DNA fragments that have adapter molecules on both ends and to amplify the amount of DNA in the library. The number of PCR cycles was minimized to avoid skewing the representation of the library. A gel purification procedure was carried out to select the fragments sized from 250 to 350 bp to produce the library for cluster generation and sequencing. The library were qualified by Agilent 2100 bioanalyzer and quantified by Qubit and qPCR. The cluster formation and sequencing were performed on the HiSeq2000 platform following the manufacturer’s standard cBot and sequencing protocols. For the multiplexing sequencing, a 100 cycles of single read 1 is used to sequence the RNA, following by a 7 cycles of index identification and 100 cycles of single read 2.

#### Sequence Analyses, Assembly, Annotation and classification

A Perl program was written to remove low quality sequences from all sequences. Then the high quality reads were assembled with Velvet_1.2.10 to construct unique consensus sequences [30] The trimed solexa transcriptome reads were mapped onto the unique consensus sequences using Bowtie Version 0.12.8 [31]. Unigenes were compared with the NCBI Non-redundant nucleotide database and Non-redundant protein database (http://www.ncbi.nlm.nih.gov/) using BLASTN and BLASTX respectively, and with the same E-value cutoffs < = 1e-5 [32], identified by sequence similarity comparison against the SWISS-PROT (ftp://ftp.ebi.ac.uk/pub/databases/swissprot) with BLAST at E values < = 1e-10. The Clusters of Orthologous Groups (COG) of proteins database was used for functional annotation [2], Kyoto Encyclopedia of Genes and Genomes (KEGG) database was used to retrieve KO information from blast result and then established pathway associations between unigene and databases [33].

#### Differentially expressed unigene detection

The aim of our research was to learn more about the salt tolerance mechanisms and sought out the functional genes participated in, so we employed edgeR to identify differentially expressed mRNAs based on their relative abundance which was reflected by counting individual read between the two libraries [34,35]. With the help of the empirical Bayes estimation, tests based on the negative binomial distribution and differential signal analysis with other types of genome scale count data, genes with a P value < = 0.01 expression ratio > = 2 or expression ratio < = 0.5 were deemed to be significantly different between the two samples. In addition, both total expressed and differentially expressed genes were used to generate cluster diagrams by Cluster3.0 (http://bioservices.capitalbio.com/xzzq/rj/3885.shtml) using the hierarchical method. The uncentered correlation and average linkage paramaters were chosen to calculate the distance in genes and samples.

#### qRT-PCR verification

The reverse transcription reactions were carried out by M-MLV reverse transcriptase (TaKaRa) following the total RNA incubated with RNase-free DNase I. The qRT-PCR was performed with SYBR Green (TaKaRa, Japan) on ABI 7500 system (Applied Biosystems, USA). The reaction processes were as follows: 40 cycles of 30 s at 95°C, 30 s at 60°C and 30 s at 72°C following the 10 min at 95°C for pre-denaturation. The detector monitors the amount of the PCR products by detecting the fluorescent dye incorporated in the products. The Ct values represent the beginning of the exponential growth of PCR products. Different template amounts have different Ct value, so they reflect the different expression levels of different genes. The classical dissociation curves signify the accuracy in each PCR reaction. Meanwhile the standard curves acquired by a 5-dilution series of cDNA templates are also used to guarantee the veracity of the detection. The expression level of 18s rRNA which we had proved to be the most stable reference gene in *Kosteletzkya virginica* under salt stress was regarded as the reference gene, so the relative expression level of the detected genes can be acquired. The final results were determined by the means of an arithmetic three independent repeated reactions. In addition, all the primers were designed by the Beacon Designer 7.0 soft program and each experiment was independently replicated at least three times.

## Results

### Characteristics of the Illumina Sequencing and *de novo* Assembly

To get a comprehensive view of *Kosteletzkya virginica* salt tolerance, two-week old seedlings of *Kosteletzkya virginica* were poured with 1/2 Hoagland and 1/2 Hoagland with the concentration of 300 mM NaCl respectively to study the high salinity responses. High-throughput RNA-seq was carried out on treatment samples (treated with 300 mM NaCl for 2h and 12h) and control sample through Illumina HiSeq 2000 platform. Duo to we opted two different time gradient treatments, correspondingly we built three cDNA libraries (CK, S1, S2). Every single length of a single Paired-End (PE) sequencing was 101bp. An overall sequencing result was displayed in Table 1.

**Table 1:** The whole sequencing statistics for *Kosteletzkya virginica* transcriptome.

Library	CK	S1	S2
Number of reads	56259578	62924188	62019535
Total length (bp)	5383026290	6023260240	5917666558
Q20 length (bp)	5157394466	5771794663	5655649575
%	95.81	95.83	95.57
Q30 length (bp)	4842142756	5419067431	5279777945
%	89.95	89.97	89.22
Number	49879742	55794878	54688826
High quality length (bp)	4772604281	5340591220	5217936840
%	88.66	88.67	88.18

**Fig 1:**
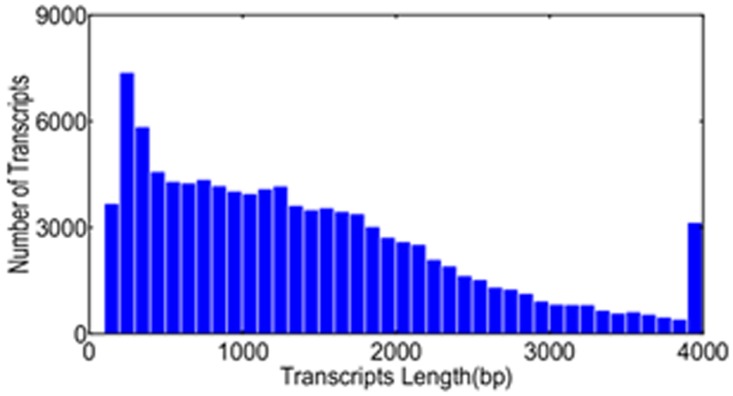
Length distribution of the assembled unigenes in the transcriptome.

When the low sequences were removed from the whole sequences by A Perl program, the high quality reads were assembled with Velvet_1.2.10 to construct unique consensus sequences [30]. Finally we got 103489 unigenes with N50 at the length of 1980 bp, average length at 1388.45 bp, the Max. length at 8972 bp and Min. length at 100 bp. The concrete distribution of the transcripts length was shown in Fig 1. In generally, there was a negative correlation between the transcripts number and the transcripts length. With the increase of the transcripts length, the number of the transcript decreased. The numbers of the reads mapped to the transcripts were 34915066 (CK), 37123677 (S1) and 40561215 (S2) reaching 70%, 66.57% and 74.17% respectively. The repository information about our transcriptome was deposited in Transcriptome Shotgun Assembly (TSA) Sequence Database with the accession number-GCJL00000000. The version described in this paper is the first version, GCJL01000000.

### Gene expression analysis

**Fig 2:**
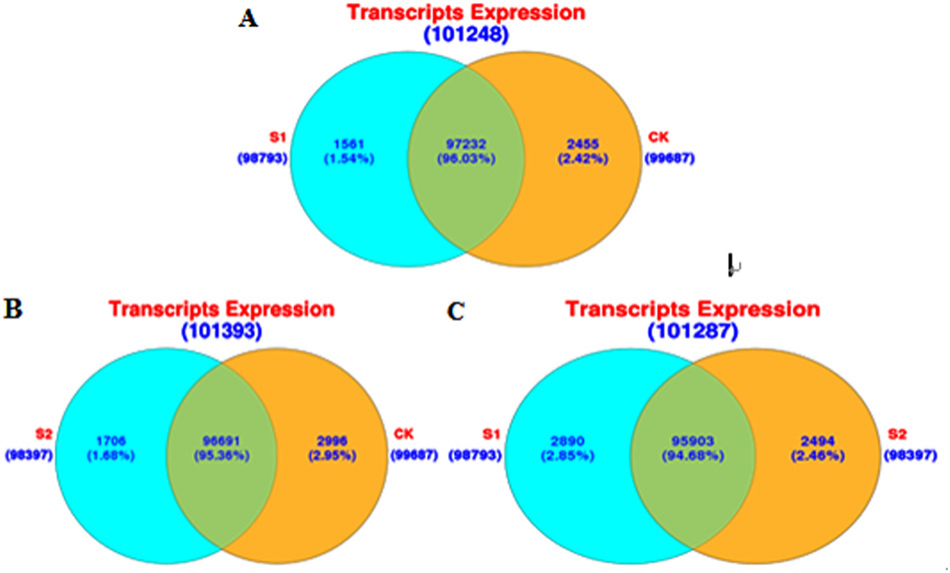
Differential expression pattern of all the transcripts in the control (CK) and two salt treatments (S1, S2) libraries.

**Fig 3:**
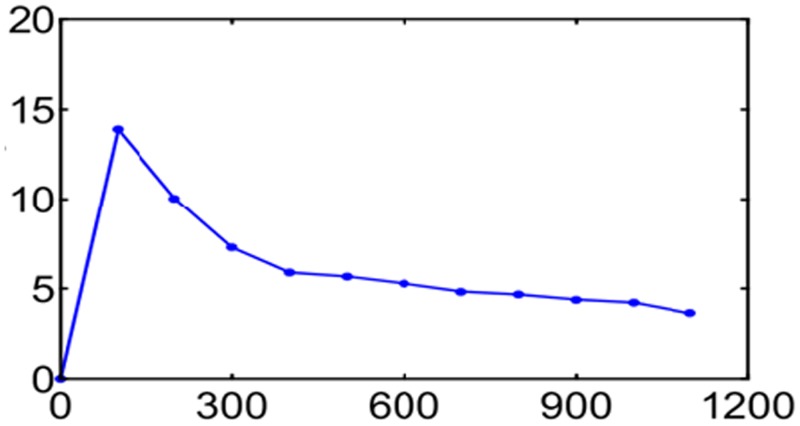
Length distribution of the predicted longest ORF.

The total transcripts expressed in three libraries were 103489, including 99687 in CK, 98793 in S1 sample and 98397 in S2. Complying with the criterion P < = 0.01, Ratio > = 2 or Ratio < = 0.5, we screened the different expressed transcripts. As shown in Fig 2A, the number of the different expressed transcripts between CK and S1 was as much as 4145, with 428 up-regulation genes and 3717 down-regulation ones. Similarly, the number between CK and S2 was 9134, with 3792 that were up-regulated and 5342 down-regulated (Fig 2B). Meanwhile the difference between S1 and S2 was 12238 including 6226 rising up and 6012 in the fall (Fig 2C). According to the central dogma, the genetic information flows from DNA by the way of transcription to RNA and arrives at protein through translation. Thus every transcript should be translated to a protein in theory and we also predicted the ORF for all the transcripts. The result demonstrated that there were 94308 ORF out of the 103489 (91.10%) total transcripts, while the remaining 9181 were not predicted. Fig 3 was the statistical diagram of the longest ORF length.

### Functional annotation and classification of all the assembled unigenes

**Fig 4:**
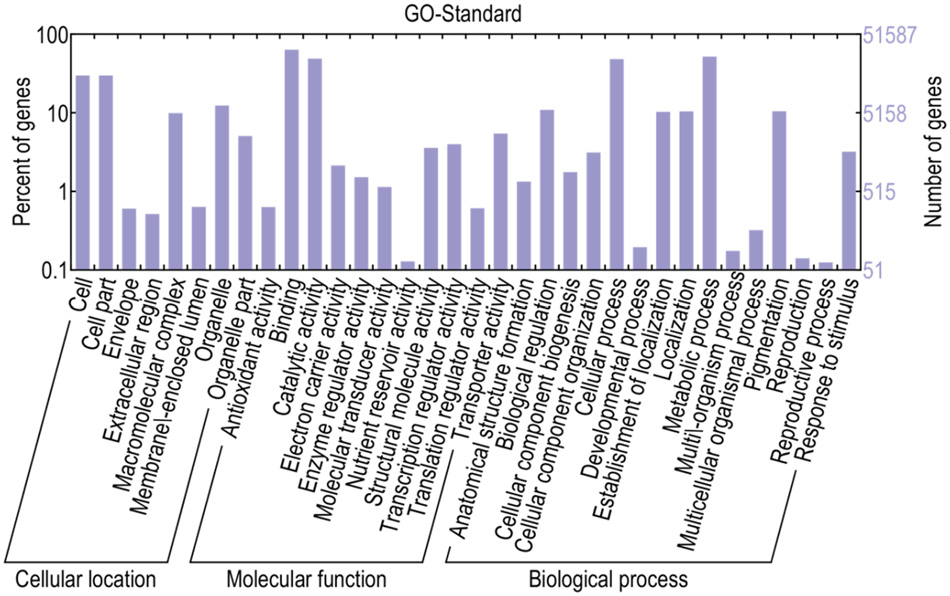
Transcripts Gene Ontology (GO) functional annotation. 51638 transcripts were assigned to GO terms and they were grouped into three main categories with 8 groups in cellular location, 11 groups in molecular function and 15 groups in biological process. The right-hand Y axis stands for the numbers of the genes in groups and the left-hand Y axis represents the percentage of the genes in categories.

It is not enough to realize only a matter of the different expressed genes. So as to know the varieties, functions and properties of the different expressed genes in different libraries, we further conducted functional annotation and classification. The GO classification and the GO trees were performed by WEGO software [36]. A total of 51638 transcripts were annotated and they were classified into 34 functional groups. The 34 functional groups were divided into three main categories (cellular location, molecular function and biological process), and the numbers of the functional groups were 8, 11 and 15 respectively (Fig 4).

**Fig 5:**
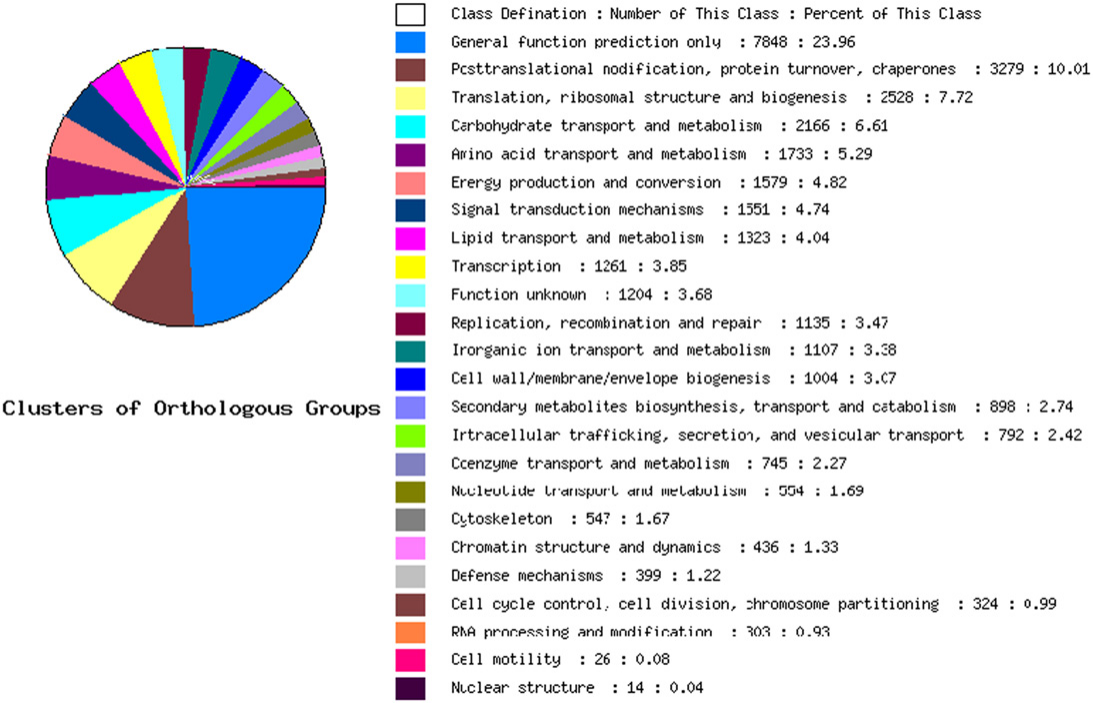
Function classification in Clusters of Orthologous Groups of proteins (COG).

**Fig 6:**
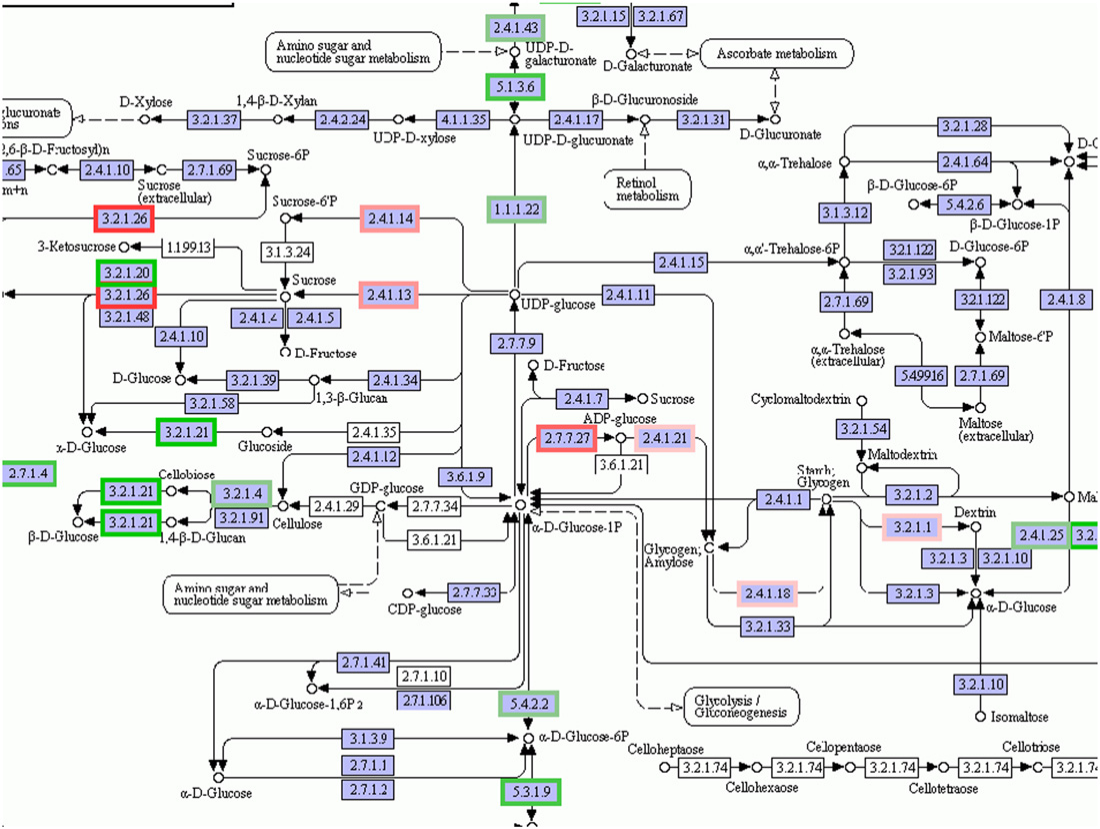
KEGG pathway visualization of starch and sucrose metabolism changes under salt stress. The enzymes circled by red frame are up-regulated in salt stress; the enzymes circled by green frame are down-regulated in salt stress in addition the more obvious variation with deeper color.

For further functional annotation, we also compared the sequence similarity against the Clusters of Orthologous Groups of proteins database (COG) with BLAST at E values < = 1e-10 [37]. 40162 transcripts were classified into 24 groups and the maximal group namely general “functional prediction only” accounted for 23.96% including 7848 genes. It was notable that the class “posttranslational modification, protein turnover, chaperones” with 3279 transcripts constituted the second largest component. In addition, there were 1204 genes belonging to “function unknown” (Fig 5). As we all know, we could learn more about the functions of the genes from the metabolism processes or signaling transduction pathways which they participated in. So unigenes were compared with Kyoto Encyclopedia of Genes and Genomes database (KEGG) using BLASTX at E values < = 1e-10 [38] for further functional prediction. In total, 82863 transcripts were located to 310 KEGG pathways which were subdivided into three sub-groups that were metabolism, genetic information processing and environmental information processing. These specific pathways provided us with more information for realizing the changes in the plants under salt stress. The primary metabolisms including starch and sucrose metabolism, photosynthesis, fatty acid metabolism, galactose metabolism, glycolysis and gluconeogenesis were influenced drastically in *Kosteletzkya virginica* under salt treatment. The KEGG pathway of starch and sucrose metabolism was shown in Fig 6.

### Differential expression analysis

**Fig 7:**
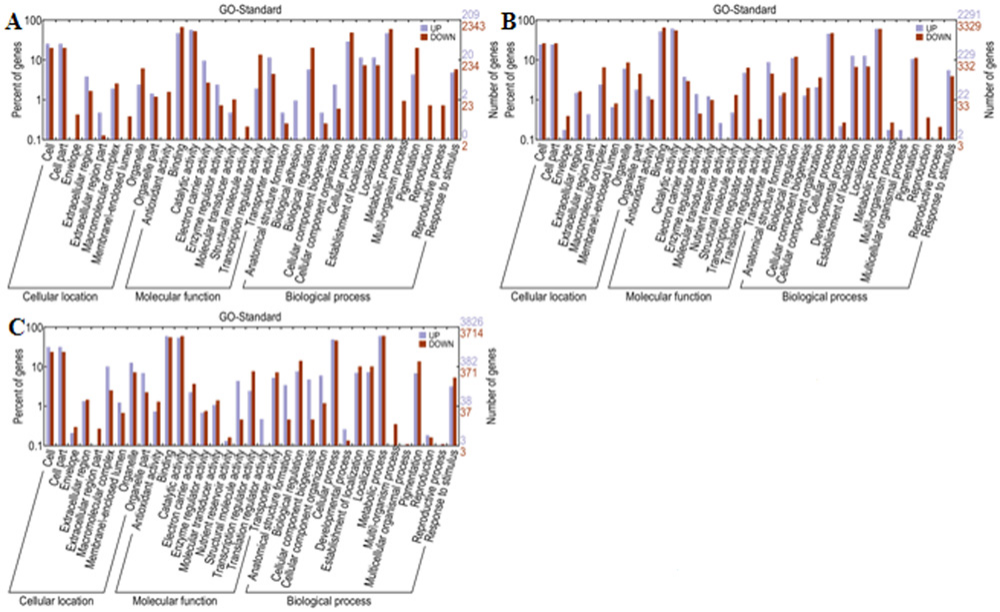
GO functional classification of the control and salt treatment seedlings. A. The comparison between S1(2h)and CK. B. The comparison between S2(12h) and CK. C. The comparison between S1 and S2.

**Fig 8:**
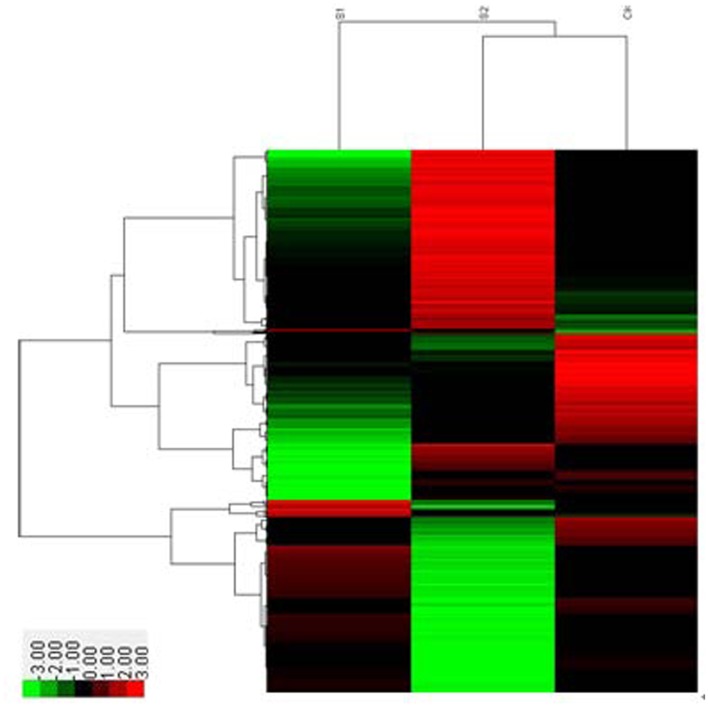
Cluster analysis of the expressed genes. Every column stands for a sample and each line represents a single gene. Different colors indicate different expression levels. Red indicates up-regulation and green indicates down-regulation, while the black indicates unchanged.

With the control and two treatment groups, we gained three cDNA libraries and performed correlated assembling and annotation. Meanwhile, we also did horizontal comparisons between pairwise samples trying to find the differences between pretreatment and after treatment as well as the differences between different treatment times. Just as we mentioned in gene expression analysis, although the large proportion of the sequenced transcripts were uniform in three libraries, the differences were also obvious and should not be ignored. And more importantly it was the minor differences that endowed the *Kosteletzkya virginica* seedlings with abilities to survive under adverse environments. The numbers of the differential expressed genes had already showed in the section of gene expression analysis. Generally genes with similar expression patterns or participating in the same pathway were functionally correlated. For further functional and correlation realization of the different expressed genes, GO, KEGG and Cluster analysis were conducted. The results of GO analysis were displayed in Fig 7A, 7B and 7C and the analysis results of the Cluster were shown in Fig 8. While the changes of the genes in KEGG analysis were labeled in different colors in every pathway so we summarized the changes of some model pathways that played important roles in salt stress in many species in Table 2. It generalized the changes of the genes in their participant pathways between control and the 12h salt tress treatment.

**Table 2:** The expression behaviors of the related genes in KEGG pathways.

KEGG Pathway	Total number of gene	Up-regulation	Down-regulation
Glycolysis / Gluconeogenesis	642	68	52
Citrate cycle (TCA cycle)	341	28	2
Starch and sucrose metabolism	531	40	50
Arginine and proline metabolism	344	66	16
Biosynthesis of unsaturated fatty acids	125	12	7
Fatty acid metabolism	206	39	10
Carotenoid biosynthesis	101	16	12
Chaperones and folding catalysts	855	41	26
Protein processing in ER	843	36	16
Transporters	82	13	3
MAPK signaling pathway	165	11	16
Plant hormone signal transduction	1039	37	84
Ion channels	51	21	1

### Verification of the RNA-seq results with qRT-PCR

**Fig 9:**
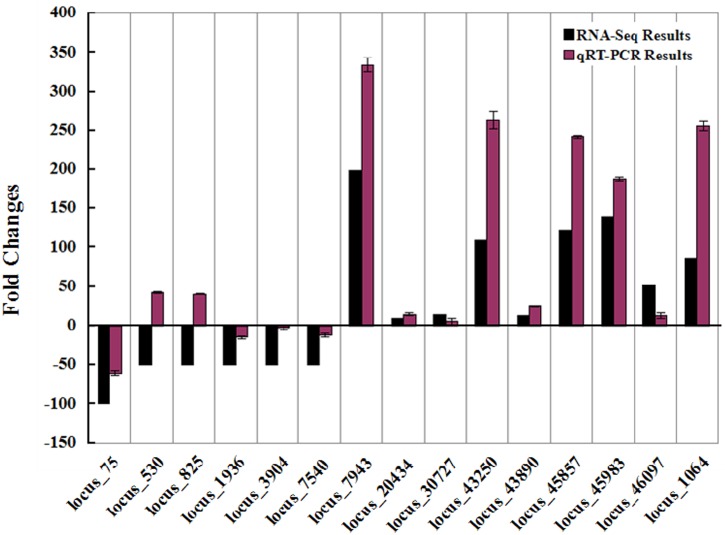
Verification of the RNA-seq results by qRT-PCR. The top ten up-regulated and five down-regulated transcripts were selected for verification by qRT-PCR. The X-axis shows the fifteen locuses and the Y-axis stands for the fold changes of the transcripts. The black bar stands for the results of RNA-seq and the red bar represents the outcomes of the qRT-PCR. The results of the qRT-PCR are mean standard deviations (± SD) of three replicates at least.

To confirm the veracity of the Illumina RNA-seq, qRT-PCR was performed with the gene-specific primers. In view of that a large proportion of the genes were up-regulated in salt stress, we chose 15 transcripts which were the top 10 up-regulated and 5 down-regulated genes in RNA-seq. The qRT-PCR was performed with SYBR Green (TaKaRa, Japan) on ABI 7500 system (Applied Biosystems, USA). The 15 genes manifesting up-regulation and down-regulation were reported to take part in abiotic stresses in many species and were involved in metabolism, signal transduction, regulation of transcription and also proteins with unknown functions. The results of the qRT-PCR and RNA-seq were as shown in Fig 9. From the figure we can see that among the fifteen detected transcripts, thirteen transcripts show the same performance while the remaining two display the opposite behaviors. Thus the results of the RNA-seq are reliable in the main. As for the differences in fold changes between qRT-PCR and RNA-seq, in our opinion the reason should be duo to their different detection methods.

## Discussion

With the development of human society, much more resources are needed to meet our human basic needs for fresh water, energy, food and feed. The United Nations Environment Program had reported that as much as 11% of the earth`s fertile soil was degraded in the latest 10 years and more over 3% would lose its biological functions in the near future [38]. Simultaneously, the recent advances in genome engineering tell us that it is possible to alter the DNA sequences precisely of a living cell to breed crops which have enhanced nutritional values, abiotic resistance or better withstand pests [39]. The experimental results of *Kosteletzkya virginica* suggested that it was superb to grow and reproduce in salt-affected coastal tidal flats (ecosystem restoration) possessing considerable genetic, environmental and economic values [40]. Continual studies on salt-resistance of *Kosteletzkya virginica* were reported, however most of the researches were merely focus on the physical signs and features while the molecular research was extremely rare [2]. Owing to the development of the sequencing, especially the second-generation sequencing technology, we conducted transcriptome sequencing on *Kosteletzkya virginica* seedlings subjected to salt-treatment (2h and 12h) and normal conditions. The high salinity condition caused radical changes in gene expression profiles of young seedlings and the different expressed transcripts were as many as thousands (Fig 2). The GO analysis further indicated that the different expressed transcripts were involved in the cellular location, molecular function and biological processes while the KEGG analysis suggested that the genes with differential expression profiles took part in the metabolism pathways, genetic information processing and environmental information processing. For example many enzymes participating in the starch and sucrose metabolism showed significant changes under our experimental treatments (Fig 7). In addition the various components such as PYR/PYL, PP2C, ABF and SnRK2 in ABA signal transduction exhibited visible changes of expression which we did not show in detail.

### Compatible solute catabolism-related genes

So as to develop and reproduce successfully, plant has evolved multifarious survival strategies on multiple levels and scales. One of the tactics that the plant adopted to resist adverse environment is the accumulation of the compatible solutes. The significance of the compatible solutes such as sugars (glucose, fructose, trehalose), free amino acids, amino acid derivatives, tertiary amines in salt stress has been well documented [41]. They not only can provide the essential energy and nutrition for plant survival under salt stress but also act as osmotic adjustment substances to balance the osmotic potential appended by high salinity [42]. For example, Poor et al. demonstrated that the accumulation of soluble sugars and sucrose was able to improve the salt tolerance [2]. Countless studies on Proline and Glycine betain also testified this point [2], and what is more the genetic engineering manipulating the metabolism of Proline and Glycine betaine acquired transgenic plants with higher stress resistance [2]. Our sequencing and analysis results also stayed the same with previous findings. As indicated in Table 2, the genes related to starch metabolism, arginine and proline metabolism, flavonoid biosynthesis and alkaloid biosynthesis changed drastically at the different time points (2h and 12h). The KEGG pathway of starch and sucrose metabolism in Fig 7 was another more visualized example displaying the up-regulation and down-regulation of the enzymes implicated in the metabolism of the soluble sugars. The cluster of orthologous groups (COG) analysis also manifested that the carbohydrate and amino acid transport and metabolism occupied a large portion of the pie arranging the third and fourth respectively (Fig 5). These statistics all indicated that the synthesis of the compatible solutes was fortified to oppose the osmotic stress in *Kosteletzkya virginica* seedlings under salt treatment. More importantly, the quantity and amplitude of expression variations at 12h were more notable than 2h and many more related genes were only manifested alterations at 12h after salt treatments.

### Genes related to phytohormone regulation

The variations of the gene expression under salt stress were also regulated by the phytohormones. As we all know, the plant hormones take part in almost all the activities of plant and the tolerance to salinity is no exception. De Bruxelles et al. had already proved that the hormones were responsible for the changes of the salt-induced genes [43]. Of all the phytohormones, ABA is the most important and its powerful functions lay in its involvement in photosynthesis, ion homeostasis, antioxidant defense and almost all the plant activities [42–45] A research on rice revealed that ABA was able to activate the activities and expressions of the CAT enzyme under abiotic stress [44] Further studies proved that ABA not only could induce the antioxidant enzymes but also could promote the accumulation of the nonenzymatic antioxidants [45]. The key component of ABA signaling factor ABI2 which was a 2C type protein phosphatase (PP2C) was discovered to bind to SOS2 and dephosphorylate it [2]. Our results also showed the same conditions. The KEGG analysis displayed that the key components of the ABA signal transduction like PP2C, ABF and SnRK2 were changed obviously under salt treatments. Moreover the AUXIN, CTK and JA signal transduction showed different degrees of responses in our research. However the SA signal transduction which was also proved to participate in salt stress in early studies did not exhibit considerable change under our experimental conditions.

### Responses of transcription factors

As has been mentioned above, the transcription factors acting as a linker between salt tolerance responses and perception can answer the high salinity sensitively and rapidly. Currently the known transcription factors responding to salt tolerance are basic leucine zipper (bZIP) [6], NAC family [10], WRKY [7], MYB [8], APETALA2/ETHYLENE RESPONSE FACTOR (AP2/ERF) and basic helix-loop-helix (bHLH) [9]. It is these transcription factors that take in charge of the expression or inhibition of their target genes and finally determine the plant salt tolerance. The MYB transcription factors were found to regulate the expression profiles of a large number of stress-responsive genes which were the so-called target genes. For instance the overexpression of the OsMYB2, a rice MYB gene, prompted the expression of proline synthetase and transporters genes as well as some other stress-related genes [46–49]. It was also in rice that the overexpression of the OsNAC6 was proved to possess enhanced drought and salt tolerance [2]. The same conclusions can also be acquired in our studies. The COG analysis (Fig 5) showed that there were as many as 1261 transcripts which were predicted to be related to transcription, indicating the highly activity of the transcription under salt stress. It is worth noting that in the top 10 up-regulated and 5 down-regulated genes up to half were predicted to be the transcription factors including MYC, MYB, NAC, WRKY and AP2/ERF, further illustrating the active responses of the transcription factors under our experimental sets.

### Other related responsive proteins

There are many other proteins that take part in salt resistance such as late embryogenesis abundant (LEA) proteins, small shock proteins (HSPs), aquaporins and so on. Their expression and synthesis are a type of self-protection strategies for plant under abiotic stresses. The LEA proteins act as osmotic protective proteins and the first LEA protein which is accumulated at the last stage of seed development is identified in cotton [47]. In recent years, the LEA proteins in many species were discovered to have high expressions under stress conditions such as drought, UV radiation, high salt, low temperatures, ethylene, and abscisic acid [2]. The transgenic sweet potato overexpressed LEA14 acquired enhanced tolerance to salt and drought [2]. We also had the similar findings and in our research the LEA proteins were up regulated as many as 140 times after 12h salt treatment indicating that in *Kosteletzkya virginica* the LEA proteins also had important roles in salt resistance. Meanwhile the analysis showed that there may be the other up regulated unknown proteins which were predicted to have the same roles and sequence information as LEA proteins. Another characteristic of our research was the significant changes of the small heat shock proteins (HSPs). As is well-known, all organisms have the abilities to accumulate HSPs under high temperature and other abiotic stresses. The HSPs which we also called molecular chaperones are involved in protein folding, assembly and transport, protecting the proteins from denaturation, promoting the mis-folded proteins to degrade and so on [2]. The key injures of the abiotic stresses to plants is the irreversible denaturation of the proteins thus the significance of HSPs is self-evident. In our research the KEGG analysis showed that the HSPs in *Kosteletzkya virginica* including HSP20, HSP70 and HSP90 which were involved in spliceosome, MAPK signaling pathway, protein processing in ER and endocytosis were changed significantly under salt stress, therefore the HSPs should play important roles in *Kosteletzkya virginica* under salt stress, too.

There is no doubt that the antioxidant enzymes also play a part in salt resistance and the KEGG analysis displays the variations of the related enzymes, while compared the variations mentioned above their behaviors are small to a certain degree.

### Conclusion

Under stress conditions, where and when the related genes are induced to express is the key for our studies on gene function analysis[48]. This study which is the first transcriptome analysis on the response of the non-model plant *Kosteletzkya virginica* reveals the holistic changes of transcription profiles on genome wide under salt stress by the NGS technology. The assembled transcript sequences represented the vast majority of the transcriptome and the comparison analysis with other known publicly available transcriptome information told us the key genes, proteins and metabolic pathways that played important roles in salt resistance. The study on halobiotic species would provide more direct and effective references for the scientific research on the molecular mechanisms of salt-tolerance, further extending our view of salt tolerance in plants. More importantly, our research would lay a foundation for future experiments on *Kosteletzkya virginica* through creating a sequence resource, opening up the research on *Kosteletzkya virginica* at the level of cell and gene.
